# TASSEL-GBS: A High Capacity Genotyping by Sequencing Analysis Pipeline

**DOI:** 10.1371/journal.pone.0090346

**Published:** 2014-02-28

**Authors:** Jeffrey C. Glaubitz, Terry M. Casstevens, Fei Lu, James Harriman, Robert J. Elshire, Qi Sun, Edward S. Buckler

**Affiliations:** Institute for Genomic Diversity, Cornell University, Ithaca, New York, United States of America; Biotechnology Resource Center Bioinformatics Facility, Cornell University, Ithaca, New York, United States of America; USDA Agricultural Research Service, Ithaca, New York, United States of America; Agriculture and Agri-Food Canada, Canada

## Abstract

Genotyping by sequencing (GBS) is a next generation sequencing based method that takes advantage of reduced representation to enable high throughput genotyping of large numbers of individuals at a large number of SNP markers. The relatively straightforward, robust, and cost-effective GBS protocol is currently being applied in numerous species by a large number of researchers. Herein we describe a bioinformatics pipeline, tassel-gbs, designed for the efficient processing of raw GBS sequence data into SNP genotypes. The tassel-gbs pipeline successfully fulfills the following key design criteria: (1) Ability to run on the modest computing resources that are typically available to small breeding or ecological research programs, including desktop or laptop machines with only 8–16 GB of RAM, (2) Scalability from small to extremely large studies, where hundreds of thousands or even millions of SNPs can be scored in up to 100,000 individuals (e.g., for large breeding programs or genetic surveys), and (3) Applicability in an accelerated breeding context, requiring rapid turnover from tissue collection to genotypes. Although a reference genome is required, the pipeline can also be run with an unfinished “pseudo-reference” consisting of numerous contigs. We describe the tassel-gbs pipeline in detail and benchmark it based upon a large scale, species wide analysis in maize (*Zea mays*), where the average error rate was reduced to 0.0042 through application of population genetic-based SNP filters. Overall, the GBS assay and the tassel-gbs pipeline provide robust tools for studying genomic diversity.

## Introduction

The advent of next generation sequencing has elicited a revolution in biology buoyed by an advancing tidal wave of raw sequence data[1]–[4]. By combining the power of next generation sequencing with reduced representation[5], which focuses sequencing resources on the ends of restriction fragments, it is now possible to quickly genotype unprecedented numbers of samples even in large genome species[6]–[8]. Several low cost, high throughput methods that combine next generation sequencing with reduced-representation have been developed (e.g., [9]–[19]). Because of its relative simplicity and robustness, the genotyping by sequencing (GBS) method of Elshire et al. [12] or close derivatives thereof have already been applied in numerous species by many researchers (e.g., [7], [17], [20]–[31]). For the first time, generation of copious quantities of genotypic data for genetic experiments is no longer a bottleneck. Instead, the new bottleneck is the efficient bioinformatics analysis of the vast and ever-expanding sea of data. Opportunities to apply markers to breeding or conservation biology are now often limited only by the availability of appropriate bioinformatics tools.

**Figure 1:**
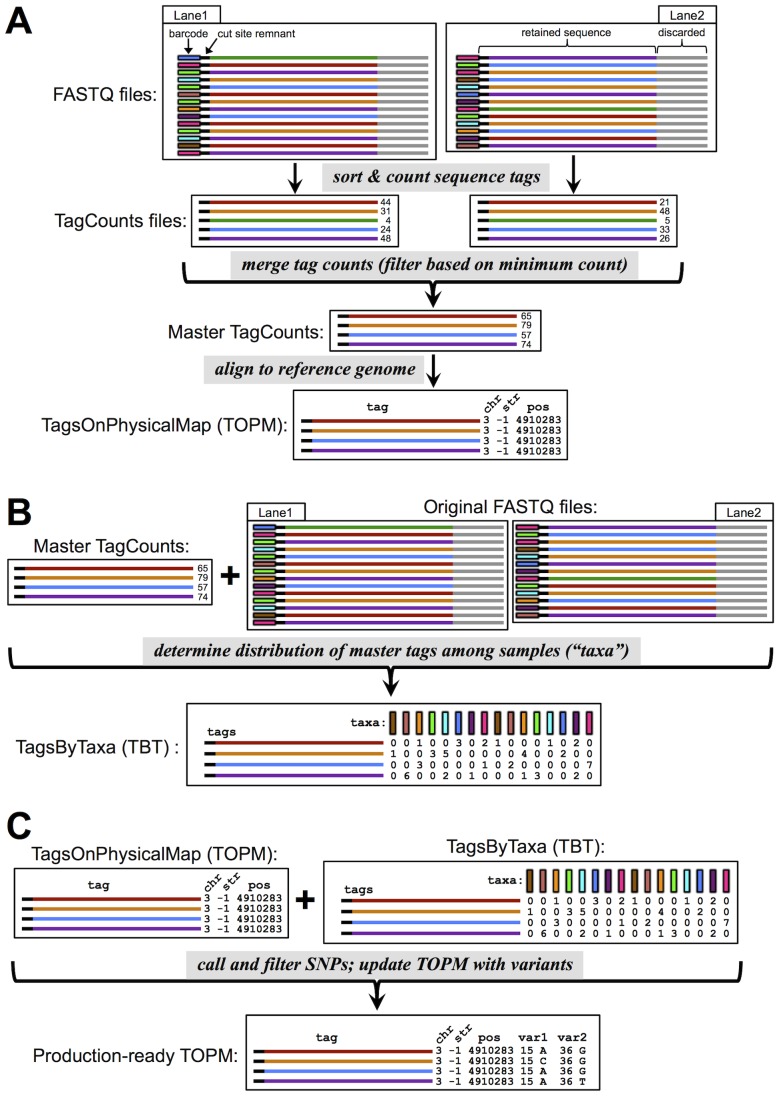
Schematic representation of the tassel-gbs Discovery Pipeline. (A) Barcoded sequence reads are processed and collapsed into a set of unique sequence tags, with one TagCounts file produced per input FASTQ file. The separate TagCounts files are then merged to form a “master” TagCounts file, which retains only those tags present at or above an experiment-wide minimum count. This master tag list is then aligned to the reference genome and a TagsOnPhysicalMap (TOPM) file is generated, containing the genomic position of each tag with a unique, best alignment. (B) The barcode information in the original FASTQ files is then used to tally the number of times each tag in the master tag list is observed in each sample (“taxon”) and these counts are stored in a TagsByTaxa (TBT) file. (C) The information recorded in the TOPM and TBT is then used to discover SNPs at each “TagLocus” (set of tags with the same genomic position) and filter the SNPs based upon the proportion of taxa covered by the TagLocus, minor allele frequency, and inbreeding coefficient (F_IT_). For each retained SNP, the allele represented by each tag in the corresponding TagLocus is recorded in the TOPM file, along with its relative position in the locus. The end product of the Discovery Pipeline is a “production-ready” TOPM that can then be used by the Production Pipeline to call SNPs.

To address this bioinformatics bottleneck, we implemented a GBS analysis pipeline in the Java program TASSEL[32] (version 4) that is specifically tailored to the GBS protocols of Elshire et al. [12] or Poland et al. [20]. However, the tassel-gbs pipeline is not limited to the specific restriction enzymes utilized in those protocols: it currently accepts 15 single restriction enzymes and 15 restriction enzyme pairs, and new enzymes are easily added. Furthermore, the tassel-gbs pipeline should work on nearly any restriction enzyme and barcoding approach (e.g., [33]), provided that sequence reads commence with the barcode immediately followed by the remnant of the restriction enzyme cut site (Figure 1A). Compared to other available pipelines for similar purposes [16], [25], [34]–[38] the tassel-gbs pipeline is specifically designed to efficiently handle large quantities of data from large numbers of samples: to date, we have analyzed more than 45,000 maize samples. The tassel-gbs pipeline was designed for species with a reference genome; however, it is possible to use incomplete genome assemblies consisting of numerous contigs as a pseudo-reference. For species without a reference genome, an alternative approach, appropriate for small to medium scale studies, has already been implemented in tassel [22].

Herein, we describe the tassel-gbs pipeline in detail, as implemented in tassel 4, and we benchmark the pipeline based upon a large-scale, species-wide analysis of maize (*Zea mays*). There were three primary motivations behind the development of this software: (1) Support the use of GBS in high-throughput, accelerated plant breeding, (2) Accommodate the high genomic diversity that is frequently encountered in species critical to agriculture and conservation, and (3) Provide an analysis platform that can be run in many contexts and with modest computational resources, such as those typically available in the developing world.

### Terminology

### A read is a single sequence in the FASTQ output file generated by the GBS assay

A ***good, barcoded read*** is a sequence read with a perfect match to one of the barcodes provided in a barcode key file and with no N's in the sequence following the barcode up to the trim length. Under the current implementation, reads are trimmed to 64 bp (not including the barcode).

A ***tag*** refers to a unique sequence (excluding the barcode) up to a specified length (currently 64 bp) from one or more “good, barcoded reads”. A given tag is typically observed in numerous good, barcoded reads of identical sequence (up to the trim length).

For our purposes, a ***taxon*** refers to a nameable entity from which one or more DNA samples can be taken.

## Design Considerations

### Separation of SNP discovery and production SNP calling

**Figure 2:**
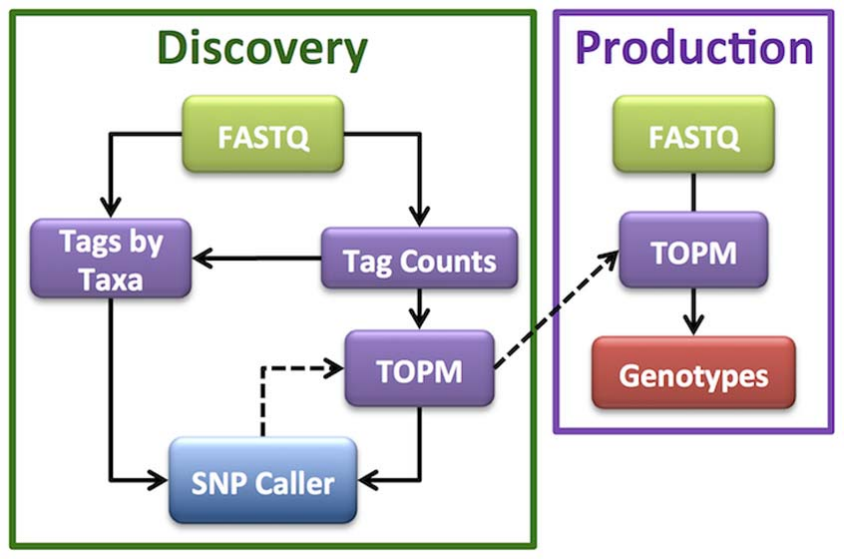
Relationship between the tassel-gbs Discovery and Production pipelines. The Discovery Pipeline is run periodically on all FASTQ files generated to date in a species, and the ascertained and filtered SNPs are stored in a “production-ready” TOPM. The Production pipeline utilizes this production-ready TOPM to quickly call SNPs either for the original samples in the Discovery Build, or for subsequent, post-Discovery samples.

Genomics-assisted, accelerated plant breeding usually consists of two separate phases of analysis: a survey of genetic diversity within a species or large breeding program to discover useful markers, followed by usage of these markers to rapidly advance generations. During the advancement cycle, time is of the essence, as thousands of samples need to be processed as quickly as possible so that decisions can be made for the next breeding cycle. The tassel-gbs software, by its division into Discovery (Figure 1) and Production pipelines (Figure 2), mirrors the two phases of accelerated plant breeding.

The aim of the Discovery Pipeline (Figure 1) is to use the cumulative sequence data from all available samples run to date in a species (or breeding population) to discover SNPs. These SNPs are stored in a “TagsOnPhysicalMap” (TOPM) data structure, containing all of the potentially useful, unique, sequence tags, the genomic positions of the subset of the tags with unique best alignment positions, and the alleles that each useful tag represents for each discovered SNP. The “production-ready” TOPM (populated with variants for each useful tag) can then be in used in the single-step Production Pipeline to quickly produce genotypes, by determining which useful tags are present in each sample. A production-ready TOPM will be applicable to a breeding population as long as the genetic diversity present in the founders of the breeding population is well represented in the individuals that comprised the corresponding Discovery Build.

Our general approach is to periodically perform a comprehensive “Discovery Build” including all samples run to date in our study species. Performing a Discovery Build on a large number of samples is a multistep process that, depending on the number of samples, can require considerable computing resources (or time). For example, the most recent Discovery Build that we performed in maize (AllZeaGBSv2.6), comprising 31,978 samples (plus 758 blank negative controls, where TE buffer was substituted in place of a DNA sample), took 495 CPU-hours on 64 core Linux machine with 512GB of RAM (where each core was a 1.4 GHz AMD Opteron Processor 6272), plus additional time for staging of all of the input FASTQ files, etc. The Production Pipeline, in contrast, provides an avenue by which genotypes for the set of SNPs discovered in the most recent Discovery Build can be quickly generated for new samples. Running the Production Pipeline on a single FASTQ file containing sequence reads from 95 or 383 samples (plus a blank negative control) requires approximately 1 CPU-hour on a MacBook Pro with a 2.6 GHz Intel Core i7 processor and 16GB of RAM running OS X.

### Favoring calling a large number of SNPs versus depth per SNP

In a GBS assay, the tradeoff, for a given genome size, between number of SNPs genotyped and the depth of coverage at each SNP is controlled by the level of multiplexing and the choice of restriction enzyme(s) [12], [20]. One of our primary motivations for performing GBS (in maize and other organisms) is to enable GWAS, which requires a high density of markers, so that each causative polymorphism stands a reasonable chance of being in LD with one or more markers [39]. Hence, we favor increasing the number of markers at the expense of depth and thus designed the tassel-gbs pipeline with low coverage data in mind. The resultant missing data and under-calling of heterozygotes can be compensated for by redundant coverage of haplotypes at high marker density, facilitating imputation.

The likelihood of success of this imputation-based strategy depends on the number and length of homozygous, identical by descent (IBD) segments present in the study population. This, in turn, depends on the demographic history of the population. Imputation is least challenging in biparental populations consisting of RILs [40]. Imputation can also be relatively straightforward in a set of homozygous inbred individuals descending from a limited number of founders (e.g., modern maize lines; [24]). Even in outcrossing species, extensive homozygous IBD stretches can be present if a population bottleneck of sufficient severity occurred at some point in the demographic history of the study population [41]. Such bottlenecks are common in outcrossing crop species, associated with domestication (e.g., [42]), modern improvement (e.g., [43]), or the development of a breeding population [44]. For unrelated (or distantly related) individuals (either inbred or outcrossed), large sample sizes improve the prospect of successful imputation: the more individuals genotyped, the more likely a given haplotype will be present in multiple individuals in homozygous form. Marker density and depth of coverage per marker need only be high enough to permit recognition of these homozygous IBD segments.

The tassel-gbs pipeline is thus optimized for low sequencing depth (0.5 to 3×) over a large number of markers in a large sample of individuals. However, it is flexible enough for the analysis of higher coverage data either from a small genome species, or from a large genome species where a lower level of multiplexing, replicate runs of the same library preps, and/or a less frequently cutting restriction enzyme (or enzyme combination) are used.

### Capacity for large numbers of markers and samples

Our analysis strategy favoring large numbers of markers scored at low depth in a large sample of individuals requires the tassel-gbs pipeline to be able to handle very large data structures (Table 1). The largest data structure is the “TagsByTaxa” (TBT) object, which records the observed depth in each individual for each potentially useful sequence tag (where “taxa” in “TagsByTaxa” refers to an individual sample from a particular GBS library prep). Our most recent Discovery Build in maize (AllZeaGBSv2.6) comprised 97.5 million potentially useful tags from 31,978 samples (plus 758 blank negative controls), with the depth for each tag in each sample recorded as a single byte (prior to compression). In order to efficiently store and retrieve data from a matrix of this size (3.2 TB of uncompressed, raw data), we used the HDF5 storage format (http://www.hdfgroup.org). We also implemented a rapid and efficient run length compression algorithm to further decrease the storage size. At low depth and with high genetic diversity (numerous sequence tags per locus), the TBT is a sparse data matrix consisting mostly of zeros; our run length compression algorithm takes advantage of this. The sequence tags themselves are stored in a binary format requiring only 16 bytes to hold 64 bases (2 bits per base), prior to compression. Thus, 64 base tags can be conveniently held in two “longs” in Java. The 64 base upper limit on tag lengths in the current implementation of tassel-gbs will be lifted in the near future, which will be helpful for study organisms with limited diversity.

**Table 1:** Size of the key data structures used by the tassel-gbs pipeline for a recent maize “Discovery Build” (AllZeaGBSv2.6).

Data Structure	Data Points	Compressed Size	Uncompressed size
Sequencing Files	4,679 Gnt^1^	3.9 TB	11.6 TB
Tags by Taxa (TBT)	3.2 trillion^2^	82.0 GB	3,198 GB
Tags on Physical Map (TOPM)	10.2 billion^3^	6.44 GB	14.0 GB

1 Giganucleotides

2 Read depths for 97,502,532 tags across 32,736 taxa (including 758 blank negative controls.

3 105 data points per tag (with each base counted as one data point) times 97,502,532 tags.

### Capability to run on modest computing infrastructure

GBS provides an unprecedented opportunity for genomic markers to be used by researchers working in numerous species, including researchers in the developing world. Many of these potential users do not have access to big memory computers or large clusters. The tassel-gbs Discovery and Production pipelines can be run on a Linux, Mac or Windows computer with 8–16 GB of RAM. The main demand with respect to the amount of RAM required is the number good, barcoded reads in a typical FASTQ file produced from GBS, which currently ranges from 200–300 million. The small memory footprint required by the tassel-gbs pipeline, in relation to its high capacity in terms of number of markers and individual samples, renders it useful to a broad array of users without access to sophisticated computing infrastructure, a target user group which may include breeders carrying out genomic selection experiments.

### Avoiding redundant alignment of identical reads

Genotyping approaches based on whole genome, rather than reduced-representation, sequencing typically align all or most of the reads produced to the reference genome prior to calling SNPs (e.g., [36]). In contrast, the tassel-gbs pipeline first collapses all of the reads into a master tag list containing all of the sequence tags present at or above a user-specified minimum count, tallied across all of the samples in the Discovery Build (Figure 1A). Each tag in this master tag list is then aligned to the reference genome. This strategy dramatically reduces the computation time devoted to alignment, and permits the use of more computationally expensive alignment algorithms. As the master tag list for our most recent maize Discovery Build (AllZeaGBSv2.6) consisted of 97.5 million tags, distilled from more than 46.8 billion sequence reads, a 392-fold reduction in computational time devoted to alignment was achieved in our case.

### Favoring allelic redundancy over quality scores

Quality scores produced by the Illumina base caller are strongly negatively correlated with position in the read. In most whole genome sequencing approaches the position of a particular SNP in different reads is essentially random. In contrast, a GBS SNP has a consistent position in each read, as all of the GBS tags from a particular genomic location (a “TagLocus”) that are used to discover and call SNPs originate from the same restriction enzyme cut site and have the same strand orientation. Consequently, the more distal SNPs in a GBS tag tend to have lower quality scores. If quality scores were used to filter GBS reads, these more distal SNPs might end up with lower depth of coverage. Furthermore, the quality scores frequently are not indicative of true quality [45], [46]. Hence, rather than using quality scores to filter out bad reads, the tassel-gbs pipeline instead relies on the number of times a given tag has been observed as an indicator of sequence quality. GBS sequence tags that occur in a minimum, user-specified, number of reads across all of the samples in a Discovery Build are deemed as potentially useful and are kept for further processing (alignment to the genome and SNP calling). Illumina quality scores are ignored, and therefore do not need to be tracked throughout the pipeline.

### Population genetic-based filtering of putative SNPs

Putative SNPs from GBS may be of low quality for multiple reasons. The sequencing error rate for a SNP may be high because of its distance from the read start and/or its immediate sequence context [47], [48]. Alternatively, paralogous sequence tags from different loci may be mistakenly aligned to a single TagLocus, resulting in spurious SNPs. To detect and filter out error-prone SNPs, the tassel-gbs pipeline relies on population-genetic parameters such as the minor allele frequency (MAF) and, in particular, the inbreeding coefficient (or “index of panmixia”), F_IT_. Filtering based upon minimum MAF can remove spurious SNPs arising solely from sequencing error. Artifactual SNPs originating from paralogous tags will tend to be excessively heterozygous and can thus be distinguished on the basis of low F_IT_.

These population-genetic filters are most powerful if a substantial proportion of the samples consist of inbred lines. Among inbred samples, both error-prone SNPs and spurious SNPs originating from paralogous tags will appear to be excessively heterozygous. The Discovery SNP caller in the tassel-gbs pipeline allows the user to specify which samples are highly inbred, and uses this subset of inbreds to calculate F_IT_ and apply the minimum F_IT_ filter. Additional, related, filters can also be applied enforcing a minimum “inbred coverage” (proportion of the inbred samples to be non-missing at the SNP) and maximum “inbred heterozygosity score” ( =  *nInbredHets*/[*nInbredsGT1ReadHomoMin* + *nInbredHets* + 0.5], where *nInbredHets* is the number of inbred taxa that are scored as heterozygous for the SNP, and *nInbredsGT1ReadHomoMin* is the number of inbred taxa with a read depth >1 for the SNP that are scored as minor allele homozygotes).

## Implementation

### Discovery Pipeline

The Discovery Pipeline consists of multiple steps, with each step (with the exception of alignment to the reference genome) being carried out by a TASSEL “plugin” that can be run from the TASSEL 4 Standalone command line interface. Detailed documentation on the function and usage of each individual plugin is available at www.maizegenetics.net/tassel/docs/TasselPipelineGBS.pdf. Rather than describe each individual plugin, here we describe the main functions of the pipeline (Figure 1) and their key features.

In order to have maximal power to discover and filter SNPs, we advocate running the Discovery Pipeline (i.e., performing a “Discovery Build”) at the species-wide level, with all samples sequenced to date, across multiple FASTQ files. Each FASTQ file contains GBS data from multiple samples distinguished by DNA barcodes at the beginning of each read [12]. We currently perform GBS at 384 plex (3072 samples per flowcell), and run each GBS library prep on one or more flowcell lanes depending on the desired sequencing depth.

#### Collapsing reads into a master tag list

The tassel-gbs Discovery Pipeline first reads through each of the available FASTQ files and generates one output “TagCount” file per input FASTQ file (Figure 1A). Each output, binary TagCount file contains a sorted list of all the unique sequence tags observed in the corresponding FASTQ file, the length of each tag in bases, and the number of times each tag was observed. This list can be used as a key-value map where the tag sequence and length together serve as the key and the corresponding tag count serves as the value. Each nucleotide is encoded in two bits allowing tag sequences to be stored in Java longs (64 bits, or 32 bases per long). Degenerate bases or N's are not permitted and quality scores are not retained. Potentially chimeric sequences are eliminated by trimming the sequence at the corresponding restriction enzyme site, if present.

After this initial pass through all of the available FASTQ files, the individual TagCounts files are merged into a single, master TagCount file containing a list of all tags of interest for a species. Only tags occurring at or above a (user-specified) minimum number of reads across all of the FASTQ files in the experiment are retained in the output master tag list. The more times a particular tag has been observed, the less likely it contains a sequencing error. The minimum tag count controls the tradeoff between the amount of sequencing errors admitted into the analysis versus the minimum allele frequency of interest. We do not expect to eliminate all sequencing errors at this step. We are usually able to filter out or correct most of them in subsequent steps of the pipeline, or in further, downstream processing customized to the biology of the study population(s). Furthermore, tags containing one or more sequencing errors can still be useful to score SNPs at other, non-error positions.

#### Alignment of tags to the reference genome

Alignment of each tag of interest in the master tag list to the reference genome is carried out with third party software. To facilitate this, the master tag list file is converted from TagCount format into FASTQ format (with fake, uniformly high quality scores). Currently, SAM format [36] output alignment files produced by the free software programs Bowtie2 [49] or BWA [36] can be read by the tassel-gbs pipeline and converted into a “TagsOnPhysicalMap” (TOPM) file that can be used for SNP calling.

The TOPM contains all of the tags present in the master TagCount file and genomic positions for the subset of tags that align to a unique best position in the genome. Retention of all of the master tags in the TOPM, even those without unique best genomic positions, affords future incorporation of additional information regarding the positions of unplaced tags, either from alternative aligners or from genetic mapping evidence. The TOPM is sorted by tag sequence and functions as a key-value map where a tag sequence can be used as a key to retrieve its corresponding physical position. It is also possible to programmatically traverse the tags within a TOPM in their physical position order through the use of an in-memory, primitive treemap. At the SNP calling step of the Discovery Pipeline (see below), the alleles represented by each useful tag at each useful SNP (“variants”) are added to the TOPM. Three file formats of the TOPM are supported: text, binary, and HDF5.

#### Determining the distribution of tags across individual samples

With a TOPM file available, the next ingredient needed to discover and call SNPs is a matrix that records the number of times each tag in the master tag list was observed in each DNA sample, which we refer to as a TagsByTaxa (TBT) file (Figure 1B). In order to maximize the capacity of our pipeline, we currently store the TBT in HDF5 format (http://www.hdfgroup.org). The HDF5 format facilitates extremely fast read and write access to large data sets. To construct a TBT, the DNA sample of origin of each good, barcoded read in the set of input FASTQ files is determined based upon its barcode. If a good, barcoded read matches one of the tags in the master tag list, its depth in the appropriate taxon is incremented in the output TBT, up to a maximum depth of 127. Since the matrix is often extremely sparse, a custom run length encoding compression algorithm was implemented that provides a high level of compression, with minimal reduction in access speed.

#### SNP discovery and initial filtering

SNP discovery (Figure 1C) is performed for each set of tags that align to the exact same starting genomic position and strand, where the starting genomic position of a tag is defined by the cut site remnant at the beginning of the tag. Such tags, originating from the same restriction enzyme cut site and with the same orientation (but not necessarily of the same length), collectively comprise a “TagLocus”. To call SNPs and ensure that indels are handled consistently, a *de novo* multiple sequence alignment of all the tags in each TagLocus is performed using the BioJava 3.0 API [50], which implements the CLUSTAL W algorithm [51]. For each SNP in the resulting “TagLocusAlignment”, the allele represented by each tag is determined and the TBT file is consulted to tally the observed depths of each allele in each taxon. The genotype of the SNP in each taxon is then determined either by a binomial likelihood ratio method of quantitative SNP calling (the default; for details see Supplementary Text S1) or, optionally, following the method of Hohenlohe et al. [52].

After genotypes are obtained for a potential SNP, initial filtering is then performed based upon user settings for minimum minor allele frequency, and for a minimum coefficient of panmixia, or inbreeding relative to the entire population, *F_IT_*, (where *F_IT_*  =  1 – *Ho*/*He*, *Ho*  =  observed heterozygosity, *He*  =  expected heterozygosity  =  2*q*(1-*q*), and *q*  =  minor allele frequency). Error-prone SNPs and spurious SNPs from paralogy often appear excessively heterozygous, with lower *F_IT_* than expected. If the user supplies a “pedigree file” that indicates the expected inbreeding coefficient (*F*) of each taxon, then only inbred taxa, with an expected inbreeding coefficient greater than or equal to the user-specified minimum coefficient of panmixia (*minF* parameter), are used in the calculation of *F_IT_*. Inbred samples, available in many crop species and model organisms, can greatly add to the power of this filter. If enough inbred samples are available, then, additional filtering of SNPs can then be optionally performed enforcing a minimum “inbred coverage” (proportion of the inbred samples to be non-missing at the SNP) and a maximum “inbred heterozygosity score” (defined above).

To illustrate the effectiveness of these population genetic-based SNP filters, as applied in our most recent maize Discovery Build (AllZeaGBSv2.6, with 31,978 samples), we focused on the subset of 5,254 samples from the maize Nested Association Mapping (NAM) population [53]. The NAM population is a series of 25 biparental, F_2_-derived RIL families all with a common female parent, the inbred line B73. Error-prone SNPs can be identified in a biparental family through their tendency, when they are in fact not segregating, to *appear* to be weakly polymorphic, but with segregation ratios significantly deviating from the 1∶1 expectation. In contrast, non-segregating SNPs in a given family that display no spurious polymorphism are free of genotypic error in that family. The availability of the 25 biparental NAM families provides tremendous power to detect error-prone SNPs, and thus to examine the effectiveness of our filters.

We used the 5,254 NAM RIL samples to estimate error rates for three alternative sets of chromosome 10 SNPs discovered in the full set of 31,978 maize samples comprising our AllZeaGBSv2.6 Discovery Build. The three sets of chromosome 10 SNPs were obtained after application of three different filtering regimes: (1) no filters other than MAF > =  0.001, (2) a minimal filter only for MAF > =  0.01, and (3) our “standard” maize Discovery Build filters of MAF > =  0.001, minimum F_IT_ in inbred samples of 0.8, inbred coverage >0.15, and inbred heterozygosity score <0.21. The SNPs were discovered and filtered based upon all 31,978 maize samples (including the NAM RILs) and their allele frequencies were then separately calculated in each of the 25 NAM families. To minimize sampling error, allele frequency was only estimated for a particular SNP-NAM family combination if at least 19 RILs in that family had non-missing genotypes for the SNP (n > =  19). Thus, by “SNP-NAM family combination” we mean a particular SNP (e.g., “S10_2918”) in a particular NAM family (e.g., “B73 × B97”) that has at least 19 non-missing genotypes, regardless of whether it is polymorphic or not within that family. Hence, for each SNP, allele frequencies were calculated in 25 or fewer families, depending on the amount of missing data in each family. NAM family-specific minor allele calls for a SNP were classified as errors if the family-specific MAF was greater than zero but less than 0.25, and the SNP significantly deviated from 1∶1 segregation in that family at *p*<0.001 (binomial test). The overall error rate for a SNP was then estimated as the total number of these error calls divided by the total number of calls for that SNP in NAM families with n > =  19 where the SNP significantly deviated from 1∶1 segregation at *p*<0.001 (including the monomorphic SNPs). A pictorial explanation of this method of estimating error rates, using a single NAM family for illustrative purposes, is provided in Supplementary Text S2.

**Figure 3:**
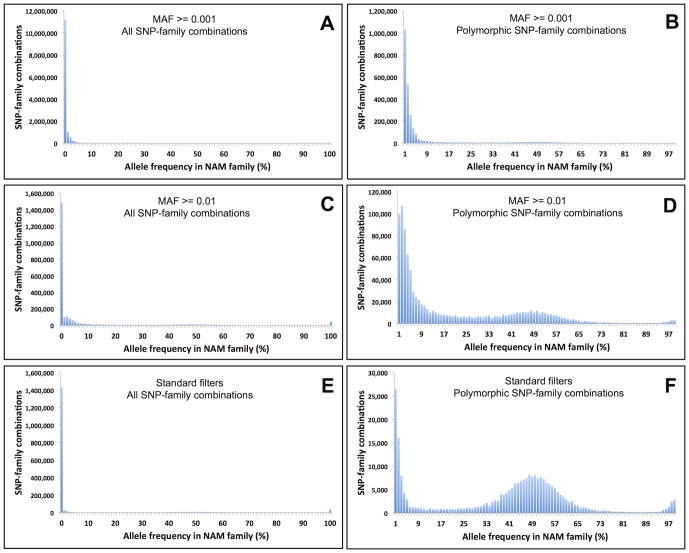
Within NAM family allele frequency distributions of chromosome 10 SNPs after different levels of filtering. Allele frequencies were calculated in each of the 25 Nested Association Mapping (NAM) families (collectively comprising 5,254 RILs) after application of the filters to the entire set of 31,978 maize samples in the AllZeaGBSv2.6 build. Allele frequencies were only estimated in a NAM family if at least 19 RILs had non-missing genotypes. Each histogram shows the allele frequency distribution for all the SNP-NAM family combinations with n > =  19. (A, B) No filter other than minimum MAF of 0.001. (C, D) A minimal filter only for MAF > =  0.01. (E, F) “Standard” maize build filters of MAF > =  0.001, minimum F_IT_ in inbred samples of 0.8, inbred coverage >0.15, and inbred heterozygosity score <0.21. (A, C, E) All SNP-family combinations: the error-free, monomorphic SNP-family combinations dwarf the segregating SNPs in all three cases. (B, D, F) Polymorphic SNP-family combinations only: omitting the monomorphic SNP-family combinations permits visualization of the remaining allele frequency distribution.

**Figure 4:**
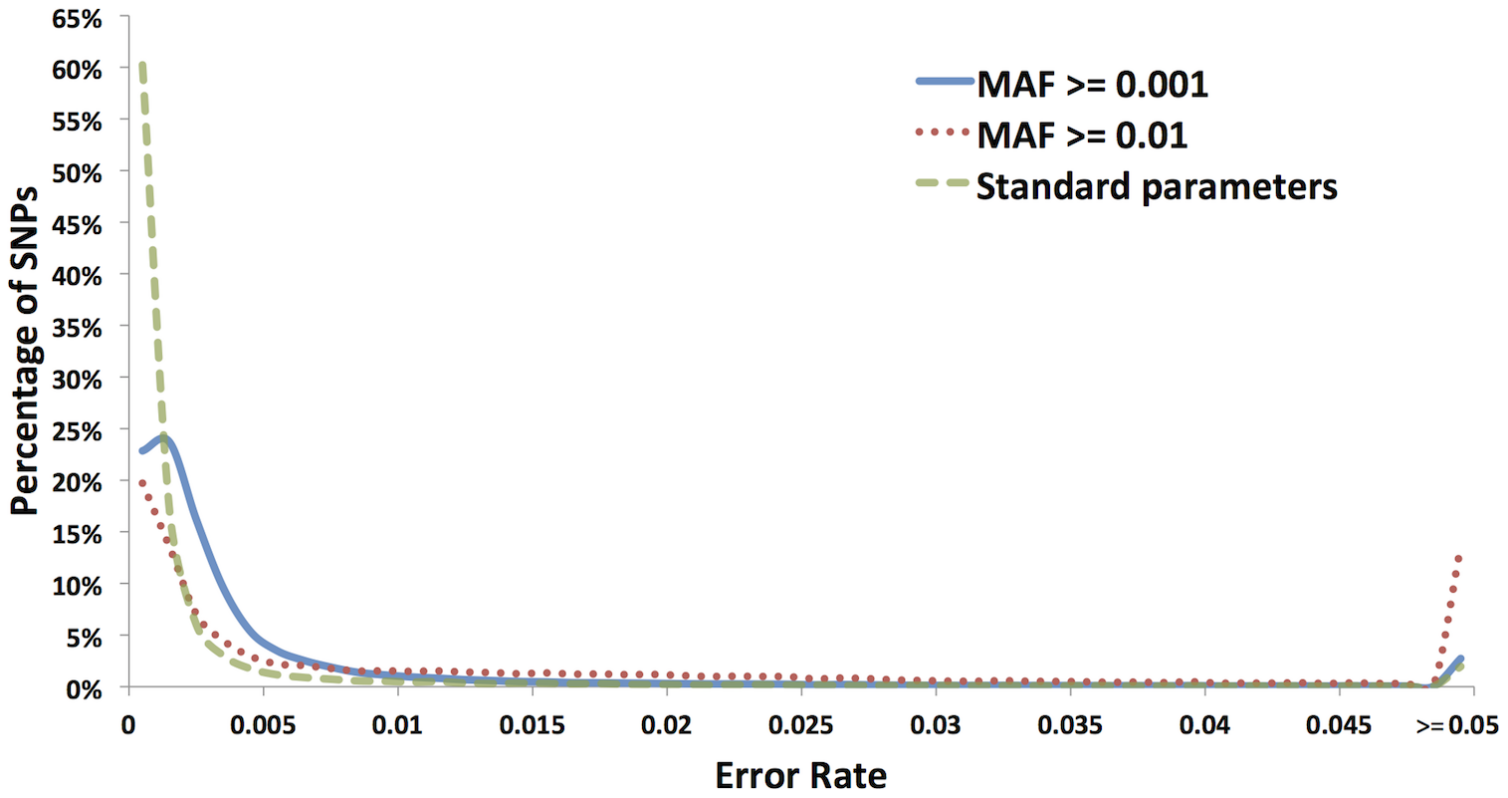
Error rate distribution of chromosome 10 SNPs for different levels of filtering. Error rates in the AllZeaGBSv2.6 Discovery build SNP calls were estimated using the NAM biparental families. NAM family-specific minor allele calls were defined as errors if the family-specific MAF was greater than zero but less than 0.25, and the SNP significantly deviated from 1∶1 segregation in that family at p<0.001.

Compared to non-filtered SNPs (Figures 3A and 3B), application of our “standard” maize Discovery Build filters (MAF > =  0.001, minimum F_IT_ in inbred samples of 0.8, inbred coverage >0.15, inbred heterozygosity score <0.21) (Figures 3E and 3F) greatly increased the proportion of SNP-NAM family combinations displaying either appropriate 1∶1 segregation (Figure 3F), or no polymorphism at all (Figure 3E). In contrast, a minimal filter based only on MAF (MAF > =  0.01) (Figures 3C and 3D) was far less effective at removing error-prone SNPs than our standard filters. Furthermore, our standard filters clearly shifted the distribution of error rates (estimated from the NAM samples) toward zero (Figure 4) and reduced the mean error rate (Table 2) relative to either no filtering (other than MAF > =  0.001) or minimal filtering (only for MAF >0.01). Extremely low estimates of mean and median error rates after application of our standard filters (0.0042 and zero respectively; Table 2) indicate that, for the most part, highly reliable SNP genotypes are produced by the GBS assay and the tassel-gbs pipeline, at least for inbred samples (where under-calling of heterozygotes due to low coverage is not an issue).

**Table 2:** Comparison of error rates for chromosome 10 SNPs from the AllZeaGBSv2.6 build for different levels of filtering by the Discovery SNP caller.

Filter^1^	nSNPs	nSNPsTested^2^	avgErrorRate^3^	avg nSegregating^4^
MAF > = 0.001	694,517	680,623	0.00681	12,899
MAF > = 0.01	149,480	136,296	0.02218	12,818
Standard^5^	78,627	78,506	0.00420	7,192

1 Filters applied to the entire build (31,978 non-blank samples)

2 Minimum sample size of 19 in at least one maize Nested Association Mapping (NAM) family

3 Average error rates estimated from 5,254 NAM RILs. Median error rates were zero for all three filters.

4 Average number of chromosome 10 SNPs with n > =  19 and MAF between 0.25 and 0.75 across the 25 NAM families.

5 MAF > =  0.001, minimum F_IT_ in inbred samples of 0.8, inbred coverage >0.15, inbred heterozygosity score <0.21.

Error prone SNPs that are not removed by our standard filters (e.g., polymorphic SNPs with family specific MAFs <0.25 in Figure 3F) can be easily removed from biparental RIL families by filtering for appropriate allele frequencies and/or based on their relatively low levels of linkage disequilibrium (LD) with neighboring SNPs. In addition, error-prone SNPs identified in biparental families can be excluded from analyses of the remaining, non-biparental samples in a Discovery Build. If biparental populations are not available in your study species, it should be possible to use half-sib (e.g., open pollinated) families, or any population having a prior expected allele frequency range for polymorphic markers, to filter out error-prone SNPs. Alternatively, recently bottlenecked populations with high levels of extended LD can be used to filter out error-prone SNPs, which should display relatively low levels of LD with their neighboring SNPs.

After SNP calling and filtering, the input TOPM is updated with variants, and this “production-ready” TOPM is then saved to a new file (Figure 1C). For each SNP that passed the filtering step, the allele that is represented by each tag in the corresponding TagLocus is recorded in the “production-ready” TOPM, as well as the relative position of the SNP with respect to the genomic position of the TagLocus. The production TOPM produced by our AllZeaGBSv2.6 Discovery Build contained 955,690 useful SNPs.

### Production Pipeline

In contrast to the multiple-step Discovery Pipeline, the Production Pipeline consists of a single step, utilizing the production-ready TOPM generated by the Discovery Pipeline to produce genotypes directly from input FASTQ files (Figure 2). The Production Pipeline determines the taxon of origin of each good, barcoded sequence read in each input FASTQ file and then checks if the read matches one of the useful tags in the production-ready TOPM. In this manner, allelic depths for each useful SNP in the TOPM are recorded for each taxon, allowing quantitative SNP calling to be performed, again either by our own binomial likelihood ratio method (for details see Supplementary Text S1) or, optionally, according to the method of Hohenlohe et al. [52]. If the GBS library preps for some samples have been run in replicate on multiple flow cell lanes (to increase depth), the corresponding allelic depth information is tallied across the replicates prior to SNP calling. Genotype files are produced in HapMap format as well as in or our own custom HDF5 format (which also records allelic depth). The ability to convert from this custom HDF5 format into VCF format [54], which also retains allelic depth, will be added to the TASSEL GUI in the near future.

### Downstream Processing

Further processing of the genotype files, such as sub-setting out specific taxa or genomic regions of interest, filtering SNPs or taxa based upon coverage, or filtering of SNPs based on minor allele frequency, can be performed either with the TASSEL 4 GUI or the TASSEL 4 Standalone command line interface (the ability to filter for SNPs that are in LD with their neighbors will be added soon). Depending on the genome size, the exact molecular protocol and restriction enzyme used, and the sequence depth obtained, missing data can be common and actual heterozygotes can be substantially under-called as homozygotes. Hence, imputation of missing data (and, possibly, phasing of the final genotypes) is usually desirable. Since the optimal imputation approach depends greatly upon the biology of the species and the experimental design, this topic is beyond the scope of this paper. Numerous, general purpose imputation tools are already available [55]. Custom imputation approaches that we are developing for our maize experimental populations will be the subject of future publications.

### Hardware Needs and Installation

The tassel-gbs pipeline is part of the TASSEL package, which is written in Java, so it can be run on Linux, Mac, or Windows operating systems. A minimum of 8 GB of RAM is required (at least 16 GB is recommended). Detailed installation instructions are provided at www.maizegenetics.net/tassel as well as in the tassel-gbs pipeline documentation (www.maizegenetics.net/tassel/docs/TasselPipelineGBS.pdf). The source code is available from sourceforge.net/p/tassel/code/ci/master/tree, JUnit tests at sourceforge.net/p/tassel/maizegenetics4-test/ci/master/tree and a relatively small test data set at www.maizegenetics.net/tassel/GBSTestData.tar. The current version of tassel-gbs as described herein is implemented in TASSEL V4.3.5. Although we generally recommend using the latest version, users can revert to V4.3.5 or other versions by following the instructions posted in the following document www.maizegenetics.net/tassel/docs/UpdatingTasselStandaloneUsingGit.pdf, under the heading “To Update Packages to Older Releases…”. The tassel-gbs pipeline will soon be available in TASSEL 5; major improvements to the pipeline (e.g., full VCF format support, allowing tag lengths greater than 64 bp, storage of tag depths per individual up to 10,000 rather than the current maximum of 127) will be implemented there.

## Strengths and Weaknesses

### Strengths

The strengths of GBS and the tassel-gbs pipeline are the large number of markers potentially produced (depending on the biology of the species and the choice of restriction enzymes), low cost and minimal startup cost, and integration of SNP discovery with SNP calling.

The potentially large number of markers available from GBS makes GWAS feasible in study populations where linkage disequilibrium (LD) extends far enough so that causative polymorphisms stand a reasonable chance of being in LD with one or more markers. Alternatively, the large number of markers facilitates accurate projection of haplotypes from a set of more densely genotyped reference haplotypes [56] derived from whole genome sequencing (WGS) (e.g., [57], [58]). This projection strategy is especially effective if the WGS reference haplotypes are representative of the founders of the study population [59].

Compared to alternative high-density marker technologies such as SNP arrays, GBS is relatively inexpensive, particularly if low coverage data suffices for the purpose of your study. Startup costs for GBS are minimal, as startup involves only (1) testing that your one of your candidate restriction enzymes (or enzyme pairs) produces a suitable GBS library, and (2) optimization of the ratio of sample DNA to the PCR adapters [12]. In contrast, startup for a SNP array involves ascertainment of SNPs in a small discovery panel and assay design for each individual SNP.

The common practice of using of a small panel of individuals to discover SNPs for inclusion in a SNP array introduces an ascertainment bias that can severely distort estimates of key population genetic parameters gauging genetic diversity, sub-population differentiation and relatedness among individuals [60]–[62]. In contrast, the tassel-gbs Discovery pipeline integrates SNP discovery with SNP calling, using all available samples to date, and thus avoids the ascertainment bias that would arise from a small discovery panel. This type of ascertainment bias will also be minimal for new samples run through the Production Pipeline, provided that their genetic diversity is well-represented among the samples included in the Discovery Build. However, there might still be some subtle biases in either pipeline, caused by factors such as null alleles [63], [64], alignment to the reference, and the use of inbreds only (rather than the full set of samples) to filter SNPs for F_IT_.

### Weaknesses

The main weakness of the GBS assay, when conducted at low coverage, is the amount of missing data. However, numerous imputation approaches are currently available [55] and yet more are currently in development, for a wide range of biological scenarios. As discussed above, the most appropriate imputation method and the probability of imputation success depends upon the biology of the study population. For some purposes, such as estimation of population allele frequencies [65], kinship, relatedness, and genetic distance, phylogenetic reconstruction [22], or germplasm quality control [24], imputation of missing data is usually not necessary.

## Conclusions

The tassel-gbs pipeline for identifying and calling SNPs from next-generation, genotyping by sequencing data fulfills our design criteria better than any existing pipeline. It has a capacity for very large analyses involving tens of thousands of samples, yet can also be run at smaller scales. The pipeline permits rapid processing of the data, yet has a relatively modest memory footprint, allowing it to be run on desktop or laptop computers. This increases its usability by researchers in developing countries who may lack access to sophisticated computing resources. The separation of SNP discovery and genotyping into two phases reduces potential ascertainment biases and, more importantly, makes the tassel-gbs pipeline highly suitable for use in a genomics-assisted, accelerated breeding context, where rapid turnaround times from tissue collection to genotypes are essential. Furthermore, the high density of markers potentially available from the GBS assay should enable accurate genomic prediction over multiple generations.

## Supporting Information

Text S1 **Description of our binomial likelihood ratio method of quantitative SNP calling.**
(PDF)

Text S2 **Estimation of GBS SNP error rates using biparental RIL families.**
(PDF)
