# Supplementary material for: TASSEL-GBS: A High Capacity Genotyping by Sequencing Analysis Pipeline
Source: PLoS One. 2014 Feb 28;9(2):e90346. doi: 10.1371/journal.pone.0090346 (PMC3938676; doi:10.1371/journal.pone.0090346)
Supplement: Text S1 — Description of our binomial likelihood ratio method of quantitative SNP calling. (PDF) [file pone.0090346.s001.pdf]

Supplementary Text S1: Description of our binomial likelihood ratio method of quantitative SNP calling

By default, the DiscoverySNPCallerPlugin performs **quantitative SNP calling** based on an expected **sequencing error rate** specified by the **-errRate option** (default of 0.01), using a binomial likelihood ratio method. Cutoffs are pre-calculated for the minimum number of reads of the "less tagged allele" needed to call a heterozygote, given the total number of reads across the two most covered alleles in a SNP in an individual taxon. These cutoffs are the *minLessTaggedAlleleCounts* such that the binomial likelihood ratio `p(Het)/p(Err) > 1`, where `p(Het)` is the binomial probability of the observed counts of the two alleles in an individual, assuming that the individual is in fact a heterozygote and that each allele is equally likely to be sequenced, and `p(Err)` is the binomial probability of the observed counts assuming that the individual is in fact a homozygote and all of the reads of less tagged allele result from sequencing errors, and that sequencing errors to the alternate allele occur at a rate specified by the **-errRate option** (default of 0.01). The cutoffs are calculated at the start, before any SNPs are called, and are written to the console output:

```
Initializing the cutoffs for quantitative SNP calling likelihood ratio
(pHet/pErr) >1

      totalReadsForSNPInIndiv  minLessTaggedAlleleCountForHet
      2                        1
      3                        1
      4                        1
      5                        1
      6                        1
      7                        2
      8                        2
      9                        2
      10                       2
      11                       2
      12                       2
      13                       2
      14                       3
      15                       3
      16                       3
      17                       3
      18                       3
      19                       3
      20                       3
      ETC…
```

From this table (which uses the default -errRate of 0.01), it can be seen that quantitative SNP calling only matters when a SNP in an individual sample has been covered by at least 7 reads in total. At less than 7 reads, it only takes one read of the less tagged allele to call a heterozygote. For a genotype covered by 20 reads in total for the two most covered nucleotides, in order for a heterozygote to be called, the less tagged allele must be covered by at least 3 reads.
