# Supplementary material for: TASSEL-GBS: A High Capacity Genotyping by Sequencing Analysis Pipeline
Source: PLoS One. 2014 Feb 28;9(2):e90346. doi: 10.1371/journal.pone.0090346 (PMC3938676; doi:10.1371/journal.pone.0090346)
Supplement: Text S2 — Estimation of GBS SNP error rates using biparental RIL families. (PDF) [file pone.0090346.s002.pdf]

Supplementary Text S2: Estimation of GBS SNP error rates using biparental RIL families

We used biparental families to detect genotyping errors. Figure 3 in the main text shows composite allele frequency distributions for chromosome 10 SNPs across the maize Nested Association Mapping (NAM) population, with each SNP-NAM family minor allele frequency treated as a separate observation. The NAM population is composed of 25 biparental families all with a common female parent (B73), and with each family consisting of >200 F2-derived RILs (expected segregation ratio of 1:1). Here (in this supplement) we use the allele frequency distribution in a single NAM family, B73 x B97, to illustrate our method of estimating error rates for GBS SNPs. It is important to understand that the SNPs were discovered and filtered using the full set of 31,978 samples in the AllZeaGBSV2.6 Discovery Build, and *then* their allele frequencies were examined in the subset of these samples consisting of the NAM RILs. Hence, a large proportion of the SNPs would not be expected to segregate in any given NAM family.

Genotyping errors are apparent in a biparental RIL family for SNPs that in fact do not segregate but *appear* to be weakly polymorphic, with family-specific minor allele frequencies outside the range expected for proper 1:1 segregation (defined in our case as a family specific MAF < 0.25 with n >= 19 and binomial $p < 0.001$). The genotypic error rate for these SNPs is their family-specific MAF. Note that the family-specific minor allele will not necessarily be the same as the minor allele with respect to all 31,978 samples (it is the latter that is plotted in the allele frequency histograms for the NAM families); with proper 1:1 segregation, which allele is the "minor allele" in a given family is a matter of chance.

In contrast, SNPs that in reality do not segregate in a family AND are completely monomorphic in that family are free of genotyping errors in that family. This is the case for the vast majority of SNPs in Figure 3 in the main text (for all three filtering levels). These zero error rate SNPs are included in our calculation of the average and median SNP error rates.

In Figure S2.1 below (next page), the allele frequency distribution for the SNPs from the AllZeaGBSV2.6 Discovery Build (discovered in 31,978 samples using our standard maize build filters) are plotted for a single NAM family, B73 x B97. To minimize sampling error, SNPs with fewer than 19 non-missing genotypes (n < 19) were excluded from the allele frequency histogram and from error rate calculations. Figure S2.1A shows the allele frequency distribution of all SNPs with n >= 19 in the NAM family B73 x B97 (196 RILs represented by 286 DNA sample/barcode combinations, with some RILs assayed twice). In this figure, monomorphic SNPs with no errors (error rate of zero) are labeled as those with MAFs of either 0% or 100% (where the minor allele was defined with respect to all 31,978 samples in the build). SNPs with MAFs between 25% and 75% are labeled as properly segregating with unknown error rates (since low frequency errors are undetectable in these SNPs). SNPs with MAFs greater than 0% and less than 25% (or greater than 75% and less than 100%) and that deviate from 1:1 segregation with a binomial p value <0.01 are assumed to truly monomorphic, non-segregating SNPs at which all the family-specific minor allele calls are genotyping errors. The error rate for one of these SNPs was estimated as the number of these erroneous minor allele calls divided by the total number of non-missing genotypes in the family. The average or median error rate was then calculated based on all SNPs with n >= 19 in the family that significantly deviated from 1:1 segregation ($p < 0.001$), *including the monomorphic ones with error rates of zero* (the vast majority). In the B73 x B97 family, the average and median genotypic error rates were 0.00214 and zero respectively.

To allow better visualization of the allele frequency distribution, only the polymorphic SNPs in the B73 x B97 family are shown in Figure S2.1B. This of course enriches for the error-prone SNPs not removed by our standard filters.

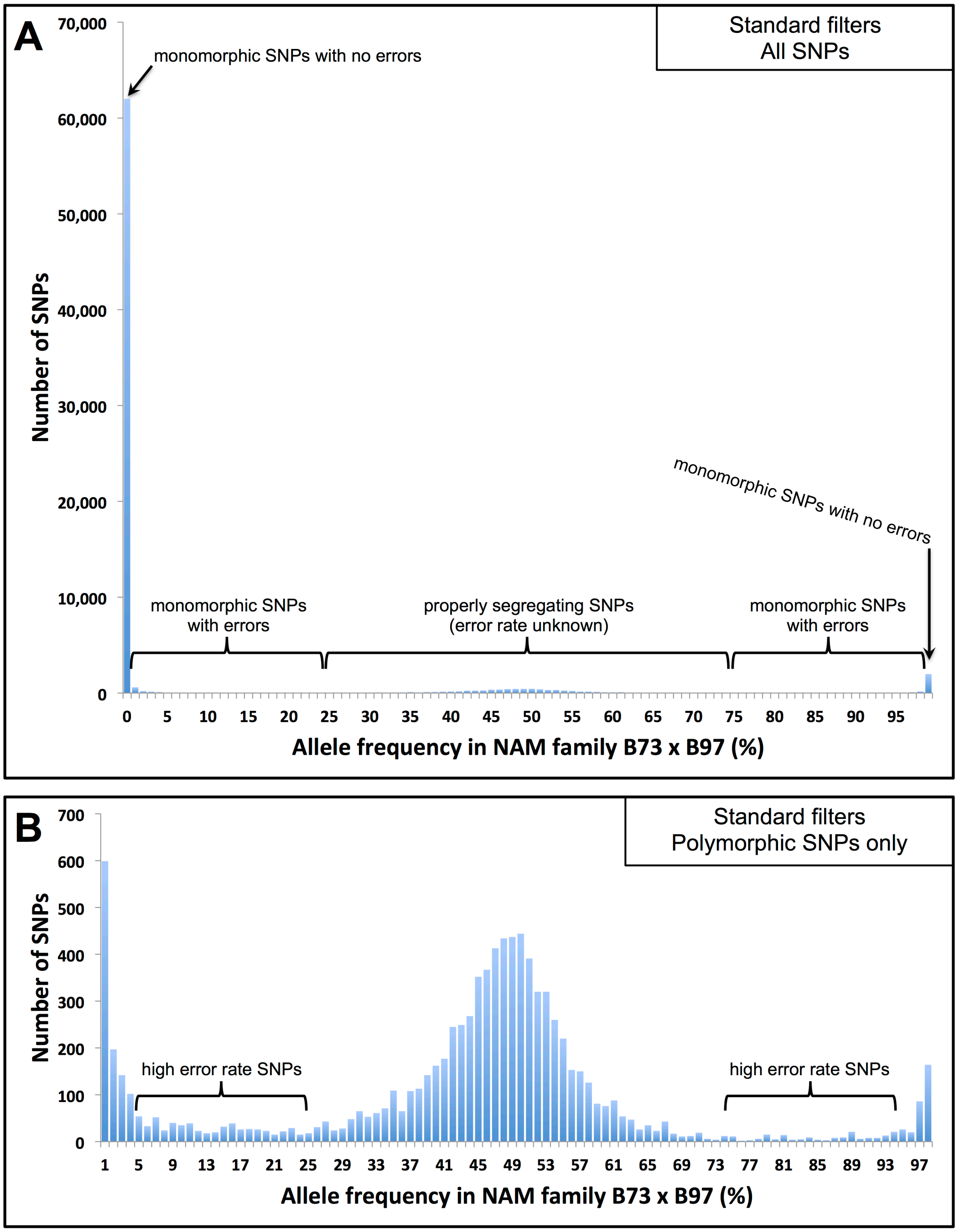

Supplemental Figure S2.1. Chromosome 10 SNP allele frequencies in the single NAM family B73 x B97 after application of our standard filters.
The standard filters (applied to the entire AllZeaGBSV2.6 Discovery Build) were MAF >= 0.001, minimum F_{IT} in inbred samples of 0.8, inbred coverage > 0.15, and inbred heterozygosity score < 0.21. (A) All SNPs with 19 or more non-missing genotypes in the NAM family B73 x B97. (B) The subset of SNPs from A that are polymorphic in B73 x B97.
